# Aquareovirus NS80 Recruits Viral Proteins to Its Inclusions, and Its C-Terminal Domain Is the Primary Driving Force for Viral Inclusion Formation

**DOI:** 10.1371/journal.pone.0055334

**Published:** 2013-02-12

**Authors:** Ling Shao, Hong Guo, Li-Ming Yan, Huan Liu, Qin Fang

**Affiliations:** 1 State Key Laboratory of Virology, Wuhan Institute of Virology, Chinese Academy of Sciences, Wuhan, China; 2 University of Chinese Academy of Sciences, Beijing, China; George Mason University, United States of America

## Abstract

Cytoplasmic inclusion bodies formed in reovirus-infected cells are the sites of viral replication and assembly. Previous studies have suggested that the NS80 protein of aquareovirus may be involved in the formation of viral inclusion bodies. However, it remains unknown whether other viral proteins are involved in the process, and what regions of NS80 may act coordinately in mediating inclusion formation. Here, we observed that globular cytoplasmic inclusions were formed in virus-infected cells and viral proteins NS80 and NS38 colocalized in the inclusions. During transfection, singly expressed NS80 could form cytoplasmic inclusions and recruit NS38 and GFP-tagged VP4 to these structures. Further treatment of cells with nocodazole, a microtubule inhibitor, did not disrupt the inclusion, suggesting that inclusion formation does not rely on microtubule network. Besides, we identified that the region 530–742 of NS80 was likely the minimal region required for inclusion formation, and the C-tail, coiled-coil region as well as the conserved linker region were essential for inclusion phenotype. Moreover, with series deletions from the N-terminus, a stepwise conversion occurred from large condensed cytoplasmic to small nuclear inclusions, then to a diffused intracellular distribution. Notablely, we found that the nuclear inclusions, formed by NS80 truncations (471 to 513–742), colocalized with cellular protein β-catenin. These data indicated that NS80 could be a major mediator in recruiting NS38 and VP4 into inclusion structures, and the C-terminus of NS80 is responsible for inclusion formation.

## Introduction

Viral replication and assembly in infected cells are commonly concentrated in specific structures either in the cytoplasm or the nucleus [1], [2], [3], [4], termed VF (viral factories), VIB (viral inclusion bodies) or viroplasms. Similar to some cytoplasmic DNA viruses (for instance vaccinia virus, iridoviruses and african swine fever virus), the replication of dsRNA viruses,such as reoviruses, also takes place in particular locations in the cytoplasm [5], [6], [7], [8]. Generally, phase dense structures in virus infected cells first appeared as numerous small granules dispersed throughout the cytoplasm, then increased in size and number, and finally formed large perinuclear factory like structures as infection proceeded [9], [10], [11], [12]. The VF has a peculiarly dense structures and could be easily identified for their highly refractile under phase-contrast microscopy [13], [14]. Recent evidences from mammalian reovirus (MRV) and avian reovirus (ARV) as well as other genera in the family *Reoviridae*, such as rotaviruses and orbiviruses, indicate that VF or VIB structures are associated with the expression of reovirus major nonstructural protein µNS or its analogues, because singly expressed µNS or its counterpart is capable of forming viroplasm-like matrix structures in transfected cells [9], [15], [16].

The viruses in genus *Aquareovirus* of the family *Reoviridae* mainly infect aquatic animals. Many of these viruses play significant role in the morbidity and mortality of aquatic populations [17], [18]. Aquareovirus is approximately 800 Å in diameter enclosing a dsRNA genome of 11 segments in concentrated core. Sequence analysis indicated that the eleven genomic dsRNA segments (named S1–S11) encode seven structural proteins (VP1–VP7) and five nonstructural proteins [19], [20]. Of the presumed twelve proteins, five nonstructural proteins (NS80, NS38, NS31, NS26 and NS16) were thought to regulate intracellular steps in viral replication. Amongst proposed seven genera of *Aquareovirus* (*Aquareovirus* A–G), the viral structural proteins in *Aquareovirus*-A and *Aquareovirus*-C have been given more attentions for understanding relationships between viral genome and protein structures and functions [20], [21], [22], [23], [24]. Phylogenic analysis and 3D structural reconstructions by Cryo-EM indicated that the similarities between aquareoviruses and orthoreoviruses in their protein sequences are accordant to their particle organization [19], [20], [21], [25]. However, the roles of nonstructural proteins played in the replication and pathogenesis of aquareoviruses remain unknown.

In addition to the similarities in viral structural proteins between genus *Aquareovirus* and *Orthoreovirus*, the two nonstructural proteins NS80 and NS38 of aquareovirus were predicted to play similar functions to its analogues, the µNS and σNS proteins of *Orthoreovirus*. Previous reports indicated that single µNS expressed in transfected cells is also capable of forming inclusions in the absence of any other viral proteins [9], [10]. Besides, it was demonstrated that µNS of MRV can recruit five core structural proteins (λ1, λ2, λ3, μ2 and σ2), the nonstructural protein σNS, and cellular clathrin to its inclusions, in addition to promoting association with cytoskeletal elements [9], [11], [12], [14], [26], [27], [28], [29]. σNS, another important nonstructural protein, is recognized to interact directly with µNS and may act as a plus single-stranded RNA (ssRNA) binding protein involved in recruiting mRNAs to VF for genomic RNA synthesis and assembly [9], [10]. The core protein μ2, the analogue of aquareoviruse VP4, known to be a microtubule-associated protein in facilitating the formation of either globular or filamentous inclusions in MRV, may play a role in determining VF morphology [10], [14], [30]. Moreover, extended investigations using siRNA or RNAi-based replication complementation and plasmid-based reverse genetics defined functional domains in µNS and μ2 proteins that are involved in viral replication [31], [32], [33]. Recently, substantial progress has been made in defining the functional domains of the orthoreovirus µNS protein that are involved in inclusion formation and interaction with other viral proteins [15], [16], [28]. Analysis of inclusion-related structures and functions revealed that four domains in the µNS C-terminus, including two coiled-coil structures, the intercoil linker region, and the C-terminal tail, are key elements required for inclusion activity [15], [16], [34]. Besides, the conserved single residues His-570 and Cys-572 in the intercoil region of µNS were shown to be strictly indispensable for inclusion formation [15], [16], [31].

The nonstructural protein NS80 of aquareovirus, encoded by segment S4, consists of 742 amino acids (80 kDa) [19], [35]. Previous investigations in our lab have identified that the factory-like structures are located in the cytoplasmic perinuclear region in aquareovirus infected cells and GFP-tagged full-length NS80 transfected cells by using transmission electron microscopy (TEM) of ultrathin-section and fluorescence microscopy [35]. These results suggested that aquareovirus NS80 may play roles in inclusion formation. More recently, we identified that NS80 could interact with itself as well as with NS38, VP4, and VP6 proteins by yeast two-hybrid (Y2H) system [36], [37]. The previous work permitted us to further study the involvement of viral nonstructural proteins in inclusion formation.

To better understand the roles of aquareovirus nonstructural proteins in viral life cycle, particularly in VF formation, we currently investigated the capability of nonstructural protein NS80 in inclusion formation in both infected and transfected cells. In this study, we detected that globular cytoplasmic inclusions were formed in infected cells, and viral proteins NS80 and NS38 colocalized in the inclusions. Besides, we identified that single NS80 can form cytoplasmic inclusions, and recruit NS38 or VP4 to its inclusions during cotransfection. The formation of inclusions in both virus-infected and NS80-transfected cells did not rely on the microtubules network. Meanwhile, by expanding the N-terminal truncation of NS80, we observed a stepwise conversion from big condensed inclusion to small nuclear inclusions, and then to diffused intracellular distribution. And we identified that the NS80 region spanning 530–742 was likely to be the smallest region required for inclusion formation. In addition, we demonstrated that the C-terminal tail region (residues 691–742, termed C-tail in this paper), the two coiled-coils plus their linker region (residues 513–690, named coiled-coil region throughout this article) were indispensable for inclusion formation. These results will contribute to a better understanding of the aquareovirus inclusion formation and lay a foundation for further investigation of the function of NS80 during viral assembly.

## Results

### Globular Viral Inclusions are Formed in Virus Infected Cells

**Figure 1:**
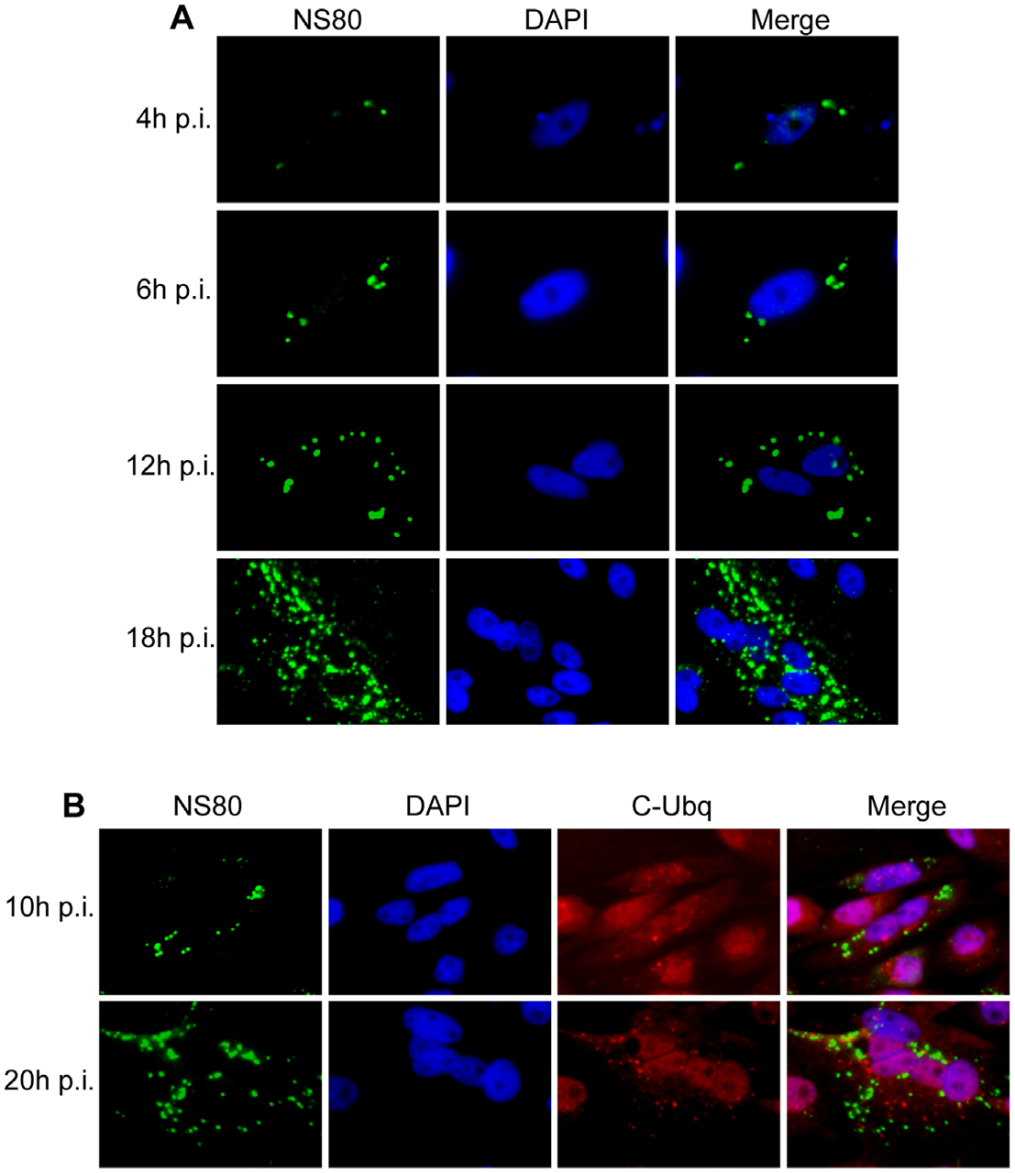
Morphogenesis of viral inclusions formed in aquareovirus infected cells. CIK monolayers were infected with 5 PFU/cell of GCRV, and then fixed at different times p.i. for immunostaining. Nuclei were stained with DAPI (blue). **A**. Time course distribution of NS80 in infected CIK cells. The cells were fixed at 4 h, 6 h, 12 h and 18 h p.i. and immunostained with rabbit anti-NS80 serum and then with FITC-conjugated goat anti-rabbit IgG (green). **B**. Ubiquitination analysis of the viral inclusions. The cells were fixed at 10 h and 20 h p.i. and then immunostained with rabbit anti-NS80 serum and then counterstained with mouse monoclonal antibody to conjugated ubiquitin, followed by FITC-conjugated goat anti-rabbit IgG (green) and Texas Red -conjugated goat anti-mouse IgG (Red).

To investigate whether viral factories or viral inclusion bodies can be formed in aquareovirus infected cells and whether the formation of viral factories is related to NS80 expression, CIK monolayers were infected with 5 PFU/cell of aquareovirus and fixed at different time post-infection (p.i.), and then immunostained with anti-NS80 polyclonal antibodies for IF microscopy. Our results revealed that NS80 was first detectable at 4 h p.i., which appeared as small immunofluorescence spots that scattered in the cytoplasm (Fig. 1A). As infection progressed, homogenous NS80 punctuates were observed and large globular perinuclear inclusions were emerged along with smaller inclusions at 12 and 18 h p.i. respectively (Fig. 1A). These structures resemble ‘viral factories’ observed in mammalian and avian reovirus infected cells [10], [12], [14]. To determine whether the inclusion structures in aquareovirus infected cells is the result of protein misfolding, the cells were immunostained with conjugated ubiquitin MAb FK2 [38] and NS80 antibodies. Our results revealed that aquareovirus VF did not colocalize with ubiquitinated proteins (Fig. 1B), suggesting that they are not formed by misfolded proteins.

### Singly Expressed NS80 can Induce Inclusion Structures

**Figure 2:**
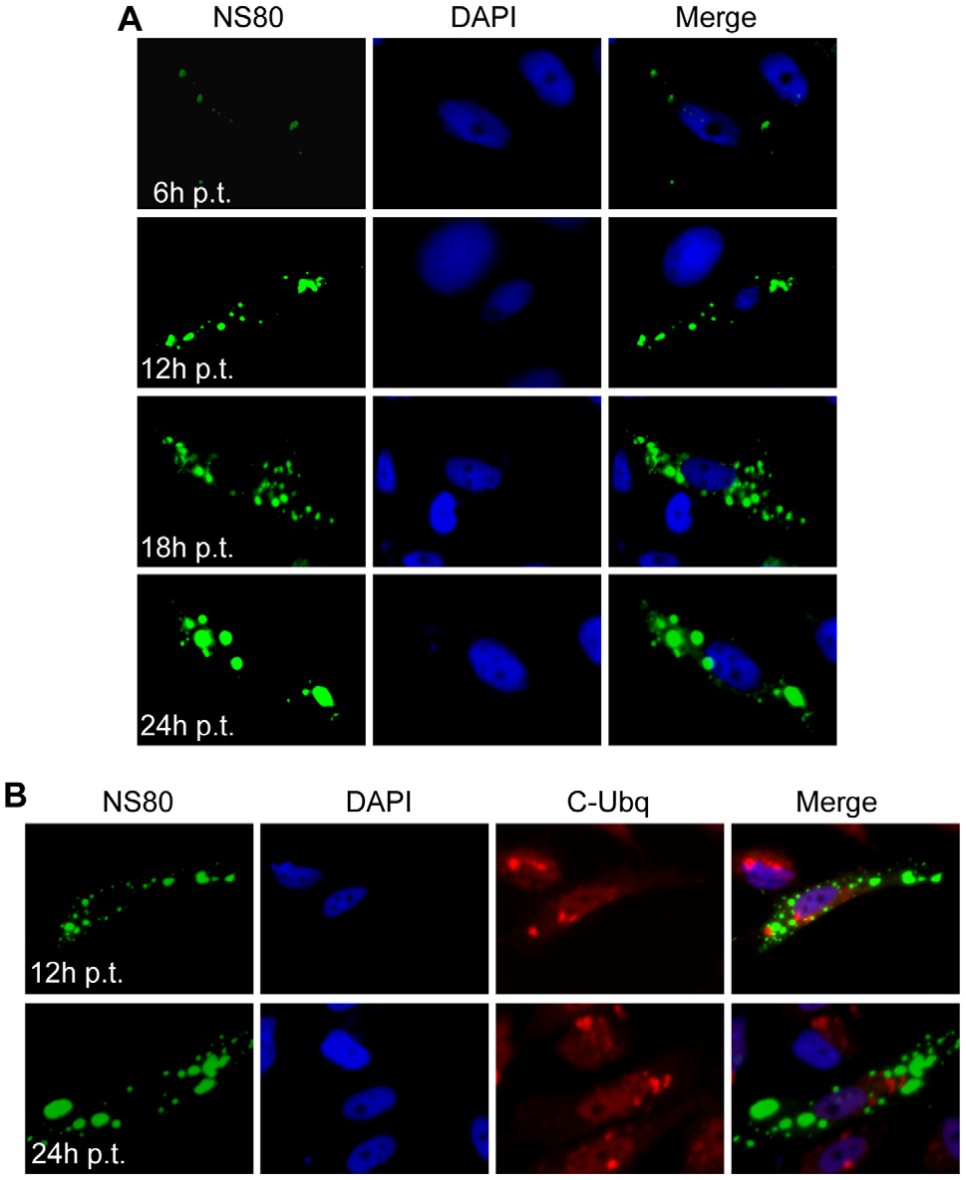
Viral inclusions formed in NS80 singly transfected cells. CIK monolayers were transfected with NS80-expressing plasmid, and then fixed at different times p.t. for immunostaining. Nuclei were stained with DAPI (blue). **A**. Time course distribution of NS80 in transfected CIK cells. The cells were fixed at 6 h, 12 h, 18 h, and 24 h p.t respectively and immunostained with rabbit anti-NS80 serum and then with FITC-conjugated goat anti-rabbit IgG (green). **B**. Ubiquitination analysis of expressed NS80 protein. The cells were fixed at 12 h and 24 h p.t. and then immunostained with corresponding antibodies.

To explore the functions of NS80 in inclusions formation, we first investigated the intracellular distribution of single NS80 in transfected cells. Plasmid expressing non-fused NS80 was transfected to different cell lines in the absence of other viral proteins. The subcellular distribution of NS80 was examined by IF at 6, 12, 18, 24 h post transfection (p.t.). Our results indicated that small or large discrete and globular phase-dense inclusions with comparatively smooth edges were observed at different times post transfection in the cytoplasm of transfected CIK cells (Fig. 2A). These inclusion structures resembled the globular phase-dense viral factories in virus infected cells. In addition, the inclusion morphology of NS80 was also detected in other transfected cell lines (Vero, L929 and Hela, data not shown), which suggested that there is no cell species-dependent for NS80 inclusion formation.

Given that misfolded proteins may accumulate in globular phase-dense structures, we utilized the polyubiquitination antibody to detect protein misfolding in NS80 inclusions. We found no colocalization of the NS80 inclusions with polyubiquitin in transfected CIK cells (Fig. 2B). These results suggested that the NS80 inclusions in transfected cells are not formed by misfolding proteins.

### NS80 and NS38 Colocalized in Viral Factories during Viral Infection

**Figure 3:**
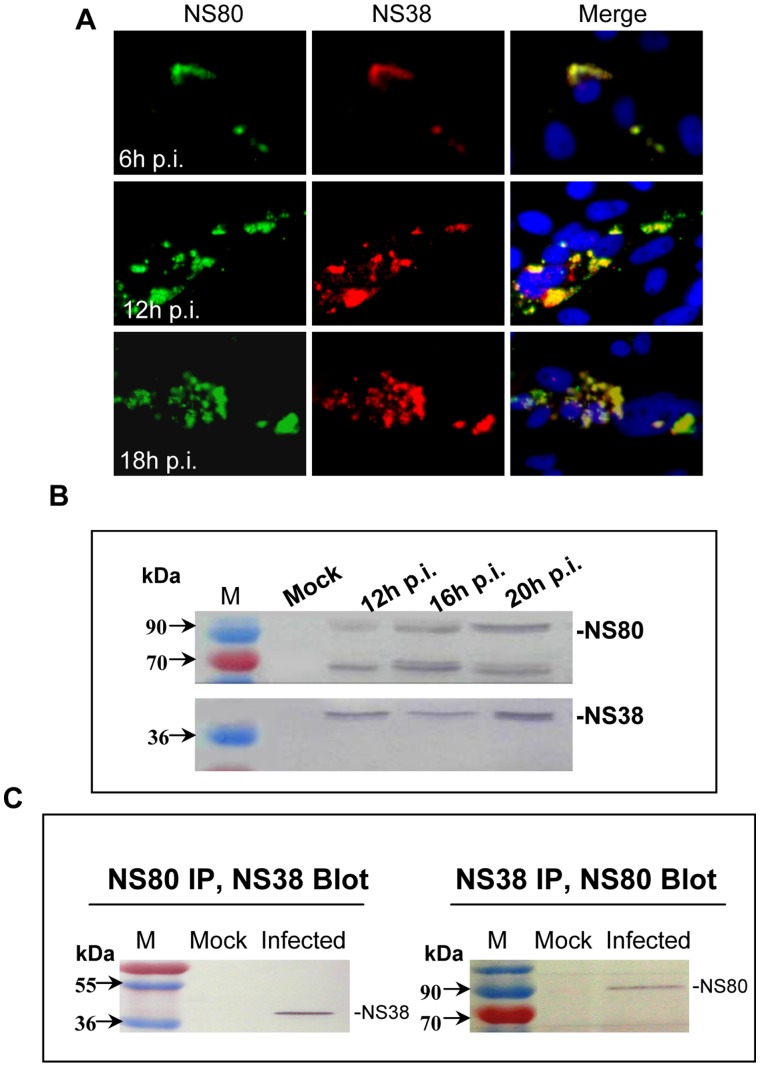
Colocalization, Western Blot and co-IP of NS80 and NS38 in aquareovirus infected cells. A . IF microscopy of GCRV-infected CIK cells at 6 h, 12 h, 18 h p.i. The subcellular localizations of NS80 and NS38 were detected by immunostaining with rabbit anti-NS80 or/and mouse anti-NS38 polyclonal antibodies followed by FITC-conjugated goat anti-rabbit IgG (green) and Texas Red-conjugated goat anti-mouse IgG (red) respectively. Nuclei were counterstained with DAPI (blue). **B**. Western Blot detection of NS80 or NS38 in virus infected cells at 12 h, 16 h, and 20 h p.i. Cell lysates were resolved by SDS-PAGE, transferred to PVDF membrane, and immunoblotted with rabbit anti-NS80 and mouse anti-NS38 polyclonal antibodies followed by alkaline phosphatase conjugated goat anti-rabbit or goat anti-mouse IgG, the images were observed by developing with AP substrate solution (NBT/BCIP). **C**. Co-IP of NS80 and NS38 from GCRV-infected CIK cells. At 20 h p.i., mock or GCRV-infected CIK cells were lysed in IP Lysis/Wash Buffer and immunoprecipitated (IP) with NS80-specific rabbit polyclonal antiserum (left) or NS38-specific mouse polyclonal antiserum (right), Immunoprecipitated proteins were subjected to Western blot analysis as indicated in Figure. 3B.

The nonstructural proteins NS80 and NS38 of aquareovirus have been implicated to be essential for viral inclusion formation during viral infection. To further identify the expression of NS38 in infected cells, we examined the subcellular localization of NS38 as well as NS80 by immunostaining with specific antibodies at different time p.i. Both NS80 and NS38 were found to colocalize in the viral inclusions (Fig. 3A). As infection progressed, NS80 and NS38 remained in the inclusions with colocalization. Further IB analysis with virus infected cell lysates confirmed the expected size of NS80 and NS38 (Fig. 3B). Moreover, a shorter polypeptide (about 70 kDa) of NS80 was also detected (Fig. 3B), which has also been found in our previous studies [35]. The colocalization of NS80 and NS38 in inclusions suggested that the two proteins may interact either directly or indirectly with each other. To further characterize the relationship between NS80 and NS38, we performed co-immunoprecipitate (co-IP) with cells lysated under nondenaturing conditions using antibodies specific either for NS80 or NS38. The co-IP result in Fig. 3C showed that, following IP with NS80-specific antiserum and IB with NS38 specific antiserum, a 38 kDa band was recognized (left); following IP with NS38 specific antiserum and IB with NS80 antiserum, an 80 kDa band was also detected in infected cells (right). These data clearly indicated that there is a direct interaction between NS80 and NS38 in infected cells.

### NS80 can Recruit NS38 to its Inclusions during Co-transfection

**Figure 4:**
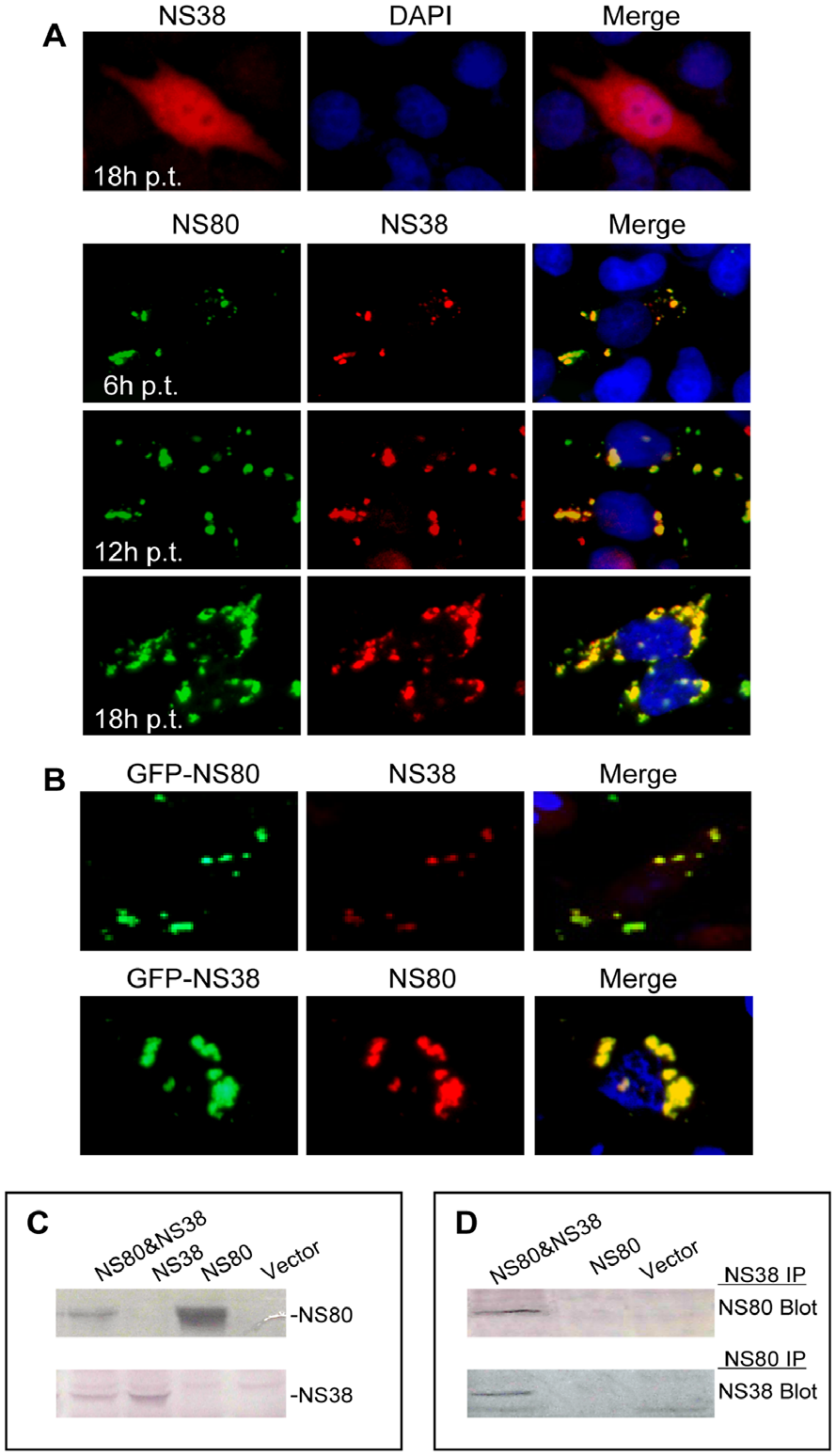
Colocalization, Western Blot and co-IP of NS80 and NS38 in transfected cells. A . IF microscopy of single transfection with pCI-neo-NS38 (up row) and cotransfection with pCI-neo-NS80 and pCI-neo-NS38 at 6 h, 12 h, 18 h p.t. The subcellular localization of NS80 was visualized by immunostaining with rabbit anti-NS80 serum followed by FITC-conjugated goat anti-rabbit IgG (green), NS38 was detected by immunostaining with mouse anti-NS38 polyclonal antibody followed by Texas Red-conjugated goat anti-mouse IgG (red). Nuclei were counterstained with DAPI (blue). **B**. IF microscopy of Vero cells cotransfected with pEGFP-C1-NS80 and pCI-neo-NS38 (top row) or pEGFP-C1-NS38 and pCI-neo-NS80 (bottom row). Cells were immunostained with corresponding antibodies. **C**. IB analysis of NS80 or NS38 in Vero cells. Cells were singly transfected or cotransfected with plasmids pCI-neo-NS80 and pCI-neo-NS38. Transfected cells were collected at 20 h p.t., lysed and analyzed by immunoblotting. **D**. Co-IP assay of NS80 and NS38 in transfected Vero cells. The transfected cells were harvested at 20 h p.t., lysed and immunoprecipitated with NS38-specific rabbit antiserum (upper panel) or NS80-specific mouse antiserum (lower panel), immunoprecipitated proteins were analyzed using IB analysis as indicated in Fig. 4C.

Subsequently, to gain a better understanding of the relationship between NS80 and NS38 in inclusion formation, we investigated the subcellular distribution of NS80 and NS38 in transfected Vero cells at different times p.t. by immunostaining. As is shown that singly expressed NS38 was diffusely distributed throughout the cells (Fig. 4A, top row), when co-transfected, NS80 and NS38 were readily detectable at 6 h p.t. and they colocalized in the cytoplasm (Fig. 4A, second row). At later time points, NS80 and NS38 still colocalized in the inclusions, as they grew in size and moved to the perinuclear region (Fig. 4A, third and bottom rows). Our results were also confirmed by co-transfecting plasmids expressing GFP-NS80 and NS38 or NS80 and GFP-NS38 into Vero cells, the coexpressed proteins all showed colocalization in cytoplasmic inclusions (Fig. 4B), indicating the GFP tag did not disturb the interaction between NS80 and NS38.

To verify the expression of NS80 and NS38 or/and their interactions in transfected cells, cell lysates were analyzed by IB (Fig. 4C). Further co-IP analyses verified that there was also a direct interaction between NS80 and NS38 in transfected cells (Fig. 4D), which is consistent with the co-IP result of NS80 and NS38 in infected cells (Fig. 3C). These results indicated that NS80 could recruit NS38 to inclusions.

### NS80 can Recruit GFP-tagged Minor Core Protein VP4 to its Inclusions

**Figure 5:**
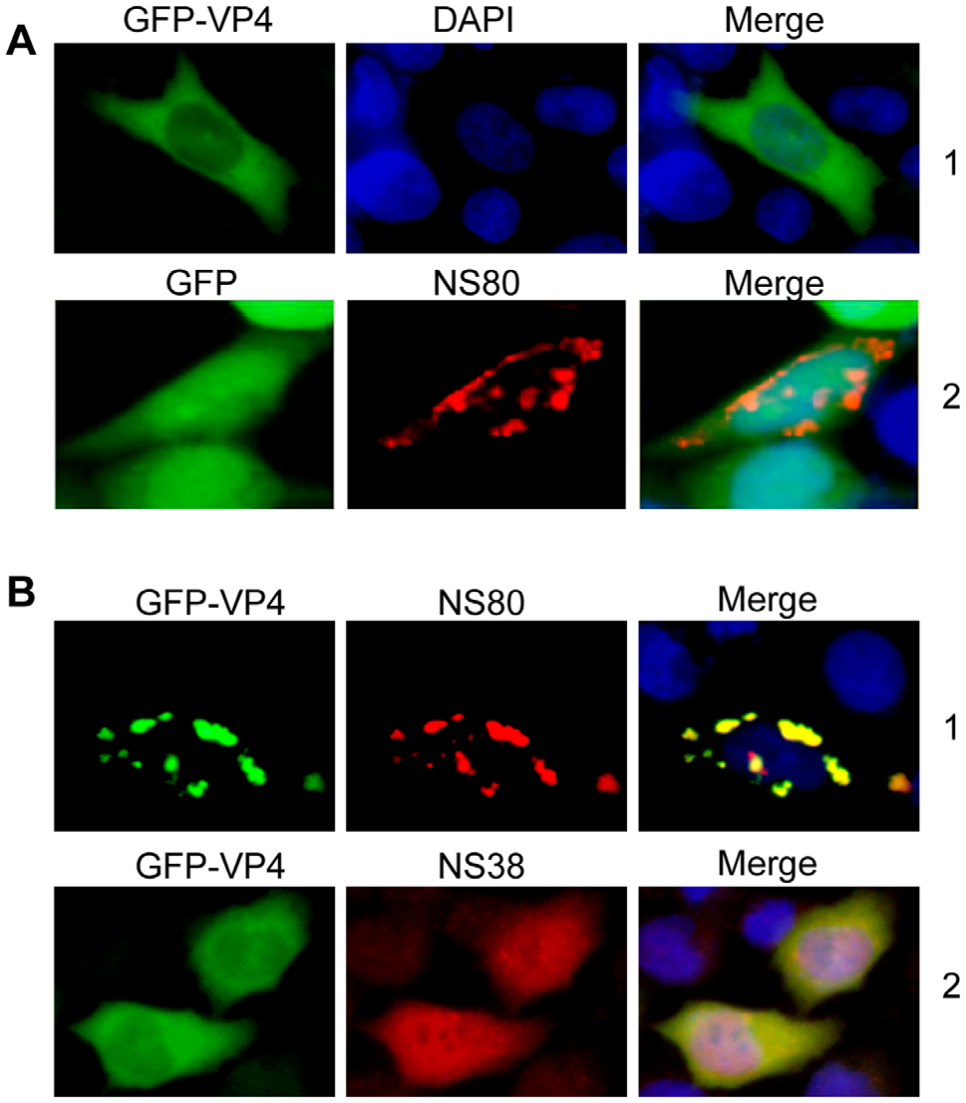
Colocalization of NS80 and GFP-VP4 in inclusion structures. A . Fluorescence microscope or IF analysis of Vero cells transfected with plasmid pEGFP-C1-VP4 (row1) or cotransfected with pEGFP-C1 and pCI-neo-NS80 (row2). **B**. IF analysis of Vero cells cotransfected with pEGFP-C1-VP4 and pCI-neo-NS80 (row 1) or pEGFP-C1-VP4 and pCI-neo-NS38 (row 2). Transfected cells were immunostained with corresponding antibodies. Nuclei were stained blue with DAPI.

To investigate the roles of another viral protein VP4 in inclusion formation and its relationship with NS80, we constructed GFP-tagged VP4 recombinant (pEGFP-C1-VP4). The plasmid was singly transfected as well as cotransfected with pCI-neo-NS38 or pCI-neo-NS80 into Vero or CIK cells. To exclude the possibility that NS80 recruits GFP to its inclusions, a control experiment was designed by co-transfecting plasmids pCI-neo-NS80 with pEGFP-C1. As shown in Fig. 5A row1 and Fig. 5B, the distribution of GFP-tagged VP4 in NS80-cotransfected cells was clearly different from that of VP4 protein expressed solely or coexpressed with NS38. No colocalization was detected with NS80 and GFP coexpression (Fig. 5A, row2). These results demonstrated that single VP4 could not form factory-like inclusion structures in transfected cells, and NS80 could recruit VP4 to its inclusions.

### The Formation of Viral Factories or NS80 Inclusions is not Dependent on Microtubules

**Figure 6:**
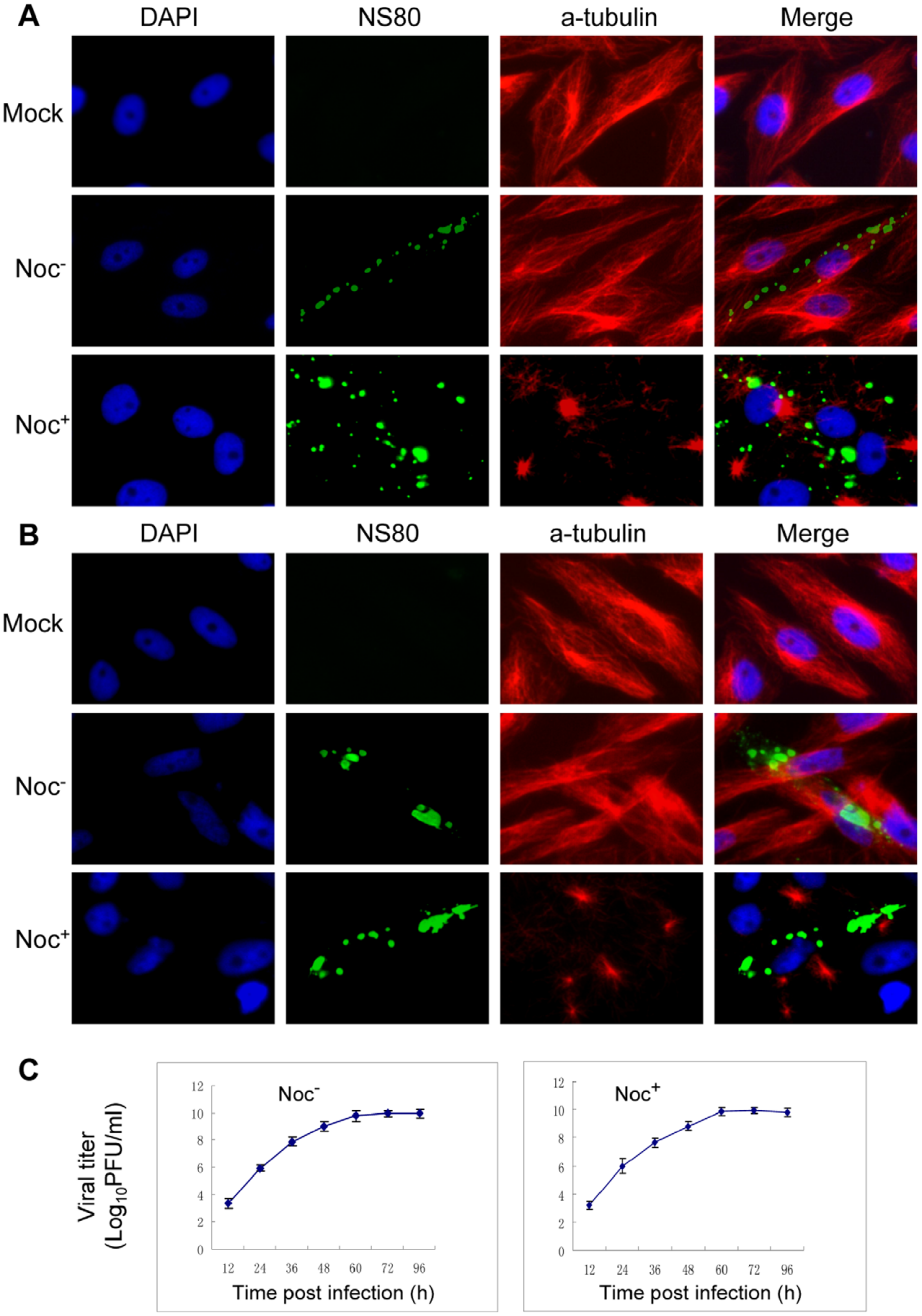
Relationships of viral factories and NS80-derived inclusions with the cytoskeleton microtubule network. A . CIK monolayers were mock-infected (row 1) or infected with 5 PFU/cell of aquareovirus (row 2 and 3). **B**. CIK monolayers were mock-transfected (row 1) or transfected with pCI-neo-NS80 (row 2 and 3). Both infected or transfected cells were either untreated or treated with 10 µM nocodazole, indicated as Noc− or Noc+. Then cells were fixed and immunostained using NS80-specific rabbit antibody and anti-α-tubulin mouse antibody followed by FITC-conjugated goat anti-rabbit IgG (green) and Texas Red-conjugated goat anti-mouse IgG (red). Nuclei were counterstained blue with DAPI. **C**. The effect of the nocodazole treatment on aquareovirus replication. Viral titers in cultured lysate stocks with nocodazole (10 µM) either untreated or treated at each time post infection were determined by PFU assays. Experiments were performed in two separated times with triple repeats, and the results are presented as the mean (±standard deviation).

To understand the basis of inclusion formation in host cells, we investigated the effects of nocodazole on the morphogenesis and intracellular localization of inclusion structures in both viral infected or NS80 transfected cells. CIK monolayers were either inoculated with aquareovirus (Fig. 6A) or transfected with pCI-neo-NS80 (Fig. 6B), and treated or mock-treated for 18 h p.i. or 18 h p.t. with nocodazole. IF analysis of the mock- or nocodazole-treated cells, using antibodies against NS80 and α-tubulin, revealed that there are no differences in inclusion morphogenesis (Fig. 6A and 6B), suggesting that the initially formed small inclusions could grow and fuse with each other to form larger one during the time of drug treatment. Furthermore, we measured viral titers from nocodazole treated or non-treated cells. As shown in Fig. 6C, titers of viral stock from nocodazole-treated cells can reach to 10^9^ PFU/ml, which was nearly the same as mock-treated cells, demonstrating that VF maturation of aquareovirus is not affected by disrupting microtubules.

### C-terminal Tail of NS80 is Necessary for Inclusion Formation

**Figure 7:**
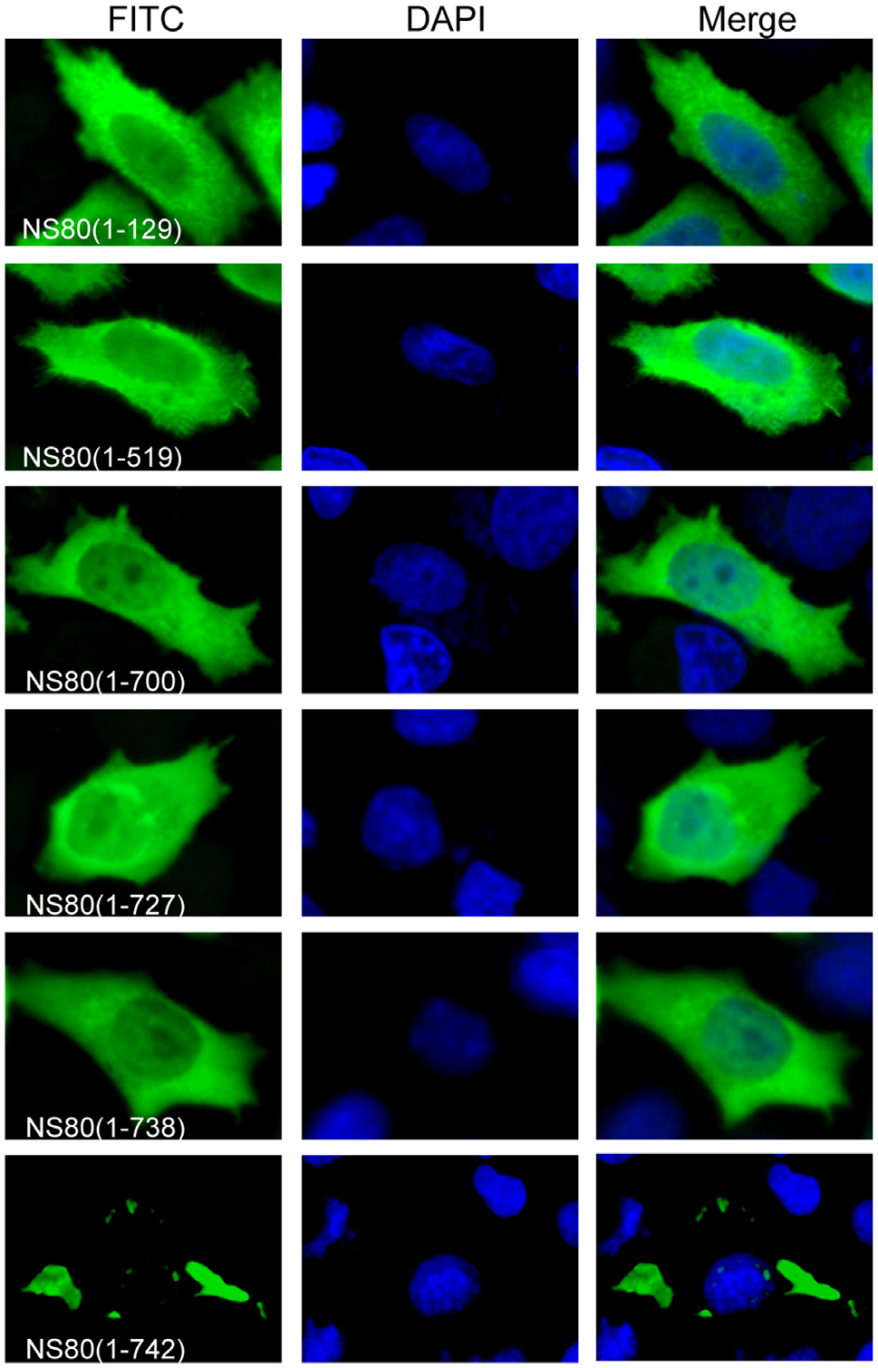
Intracellular distribution of NS80 C-terminal truncations in transfected cells. Vero cells were transfected with plasmids expressing the NS80 or its C-terminal truncations. At 18 h p.t., cells were fixed and immunostained with corresponding antibodies. Nuclei were stained blue with DAPI.

To identify functional regions of NS80 related to inclusion formation, we constructed a series of NS80 C-terminal truncations. Each of these truncations was transfected into CIK or Vero cells, and the cell lysates were subjected to IB analysis with anti-NS80 serum to confirm its correct expression (data not shown). For IF analysis, the transfected cells were fixed and immunostained with anti-NS80 antibody. As shown in Fig. 7, each of the C-terminal truncations was diffusely distributed throughout the cytoplasm and the nucleus in transfected Vero cells (for CIK cells, data not shown), in contrast to full-length NS80 which appeared in globular inclusions. The results showed that deletion of only 4 amino acids (aa) resulted in the loss of the inclusion-competent phenotype (Fig. 7), indicating that the C-tail region is indispensable for inclusion formation. Ubiquitination analysis showed that the expressed NS80 truncations were not misfolded (data not shown). As such, all of these truncated proteins can be defined as negative for forming factory-like inclusions.

### Residues 530 to 742 of NS80 is the Minimal Region Required for Inclusion Formation

**Figure 8:**
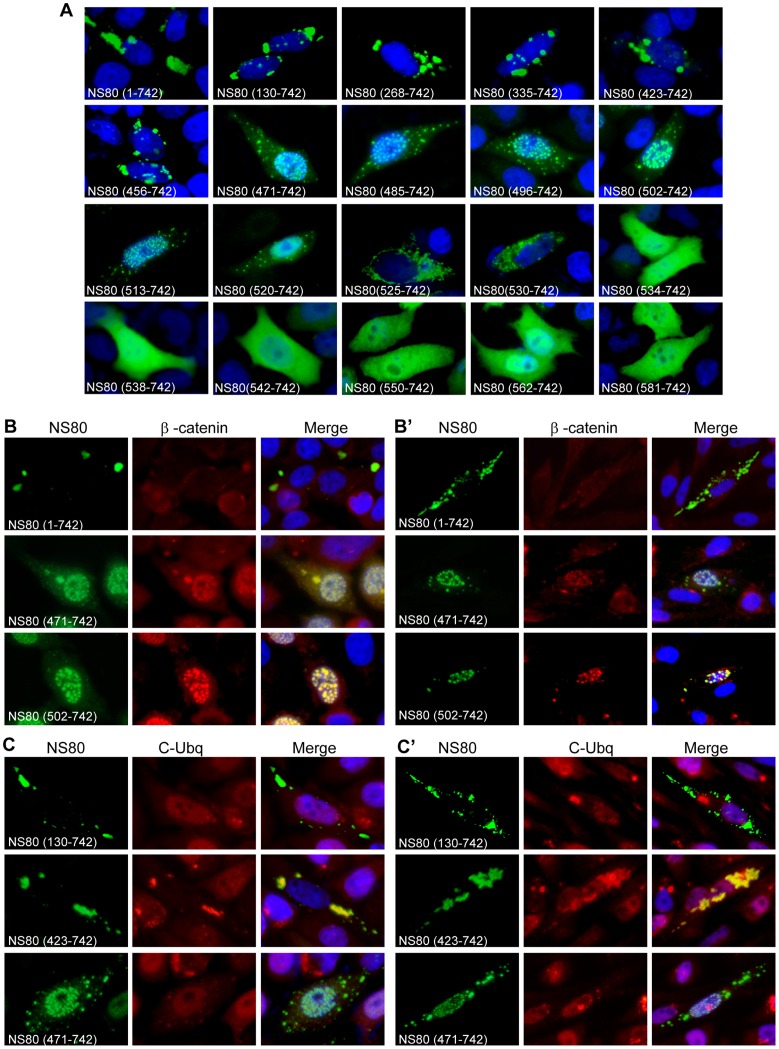
Intracellular distribution of NS80 N-terminal truncations and their relationships with cellular proteins. A. Vero cells were transfected with plasmids expressing the indicted NS80 N-terminal truncations, at 18 h p.t., cells were fixed and immunostained with rabbit anti-NS80 antibody and followed with FITC-conjugated goat anti-rabbit IgG (green). **B** and **B’**. Colocalization assay of NS80 and its N-terminal truncations with β-catenin. Vero(**B**) and CIK(**B’**) cells were transfected with plasmids expressing NS80(1–742), NS80(471–742) and NS80(502–742). Then cells were fixed and immunostained using mouse anti-NS80 and anti-β-catenin antibodies followed by FITC-conjugated goat anti-mouse IgG (green) and Texas Red-conjugated goat anti-rabbit IgG (red). **C** and **C’**. Ubiquitination analysis of N-terminal truncations of NS80 in transfected cells. Vero (**C**) and CIK (**C’**) cells were transfected with plasmids expressing NS80(130–742), NS80(423–742) or NS80(471–742). The cells were immunostained with rabbit anti-NS80 antibody and then counterstained with mouse monoclonal antibody to conjugated ubiquitin, followed by FITC-conjugated goat anti-rabbit IgG (green) and Texas Red-conjugated goat anti-mouse IgG (Red). Nuclei were counterstained blue with DAPI.

Previously, we proposed that the residues 513 to 742 of NS80 protein might be the smallest region that is necessary for forming inclusions [36]. To verify this hypothesis, we constructed 6 truncations with deletions ranging from 100 to 600 aa from the N-terminus: they are NS80(130–742), NS80(268–742), NS80(335–742), NS80(423–742), NS80(513–742) and NS80(581–742). IF observation showed that the 6 truncations represented three distinctive phenotypes (Fig. 8A). Expression of the truncations NS80(130–742) and NS80(423–742) showed typical inclusion phenotype in transfected Vero cells. And the expression of NS80(513–742) did not adversely affect its capacity to form inclusions. However, the inclusion size and location were altered a lot with small inclusions distributed mainly in the nucleus. At the same time, the truncation of NS80(581–742) presented dispersed distribution all over the cells. To further determine the minimal regions of NS80 required for inclusion formation, we further dissected the region 423–581 with extended deletions and shorter truncation distance. In this way, NS80 (456, 471, 485, 496, 502, 520, 525, 530, 534, 538, 542, 550, 562–742) truncations were constructed. IF results demonstrated that NS80(456–742) showed regular cytoplasmic inclusions. Specially, the NS80(471–742), NS80(485–742), NS80(496–742), NS80(502–742) truncations (also termed NS80(471 to 502–742)) formed small nucleic inclusions (Fig. 8A) similar to previously described NS80(513–742). We also investigated whether the nucleic inclusion is associated with cellular factors. Indeed, cell protein β-catenin was detected and found to colocalize in the nucleic inclusion with NS80 truncations from residues 471–513 to 742, but the relatively low expression of β-catenin is observed with full length NS80 in both transfected Vero and CIK cells (Fig. 8B and B’).

**Figure 9:**
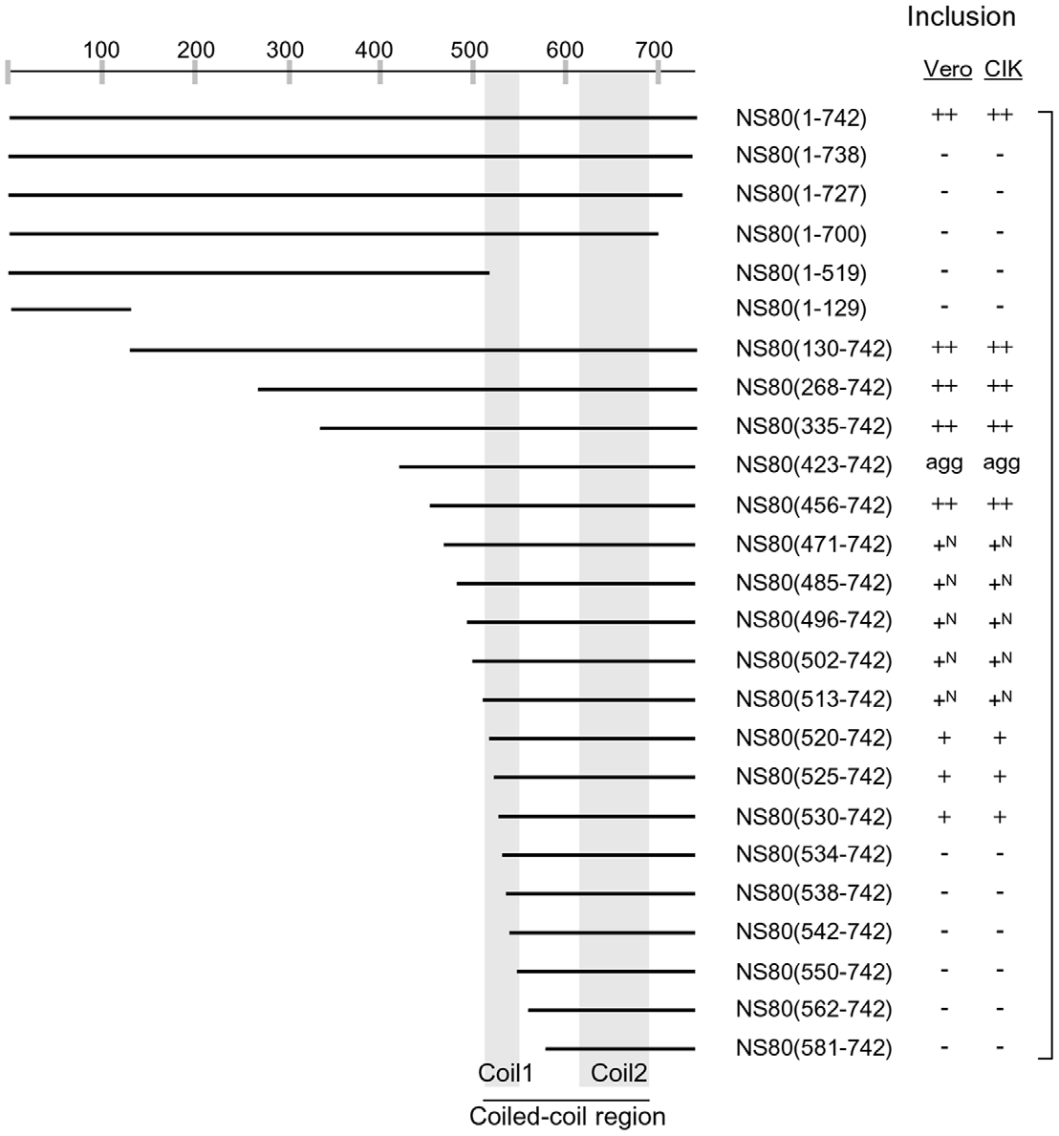
Summary of non-fused NS80 truncations and their activities for forming inclusions. The NS80 protein is indicated by a horizontally elongated black bar spanning residues 1 to 742 (positions numbered above). The NS80 truncations are also shown as black bars representing the corresponding portion of NS80. Approximate residue ranges of the predicted coiled-coil segments are indicated by vertically elongated gray bars. The capacity of expressed protein to form factory-like inclusions in transfected CIK or Vero cells was indicated on the right (−: inclusion negative/diffused; +: inclusion small; ++: inclusion intense and large; agg: aggregated). “N” indicates typical nuclear inclusions.

It is worth mentioning that the nucleic inclusions and cytoplasmic sporadic inclusions formed in NS80(471–742) transfected Vero and CIK cells did not colocalize with the polyubiquitin (Fig. 8C and C’), indicating that the nucleic inclusions were not formed by misfolded proteins. Furthermore, all the truncations were co-immunostained with conjugated ubiquitin, and we found that only NS80(423–742) was recognized by anti-ubiquitin antibodies (Fig. 8C and C’, row2). The schematic diagram of summarized results related to non-fused NS80 truncations and their activities for forming inclusions is shown in Fig. 9. In conclusion, the region spanning residues 530–742, identified by serial deletions from the N-terminus, was likely to be the minimal region required for inclusion formation.

### The Conserved Intercoil Region of NS80 is Critical for Inclusion Formation

**Figure 10:**
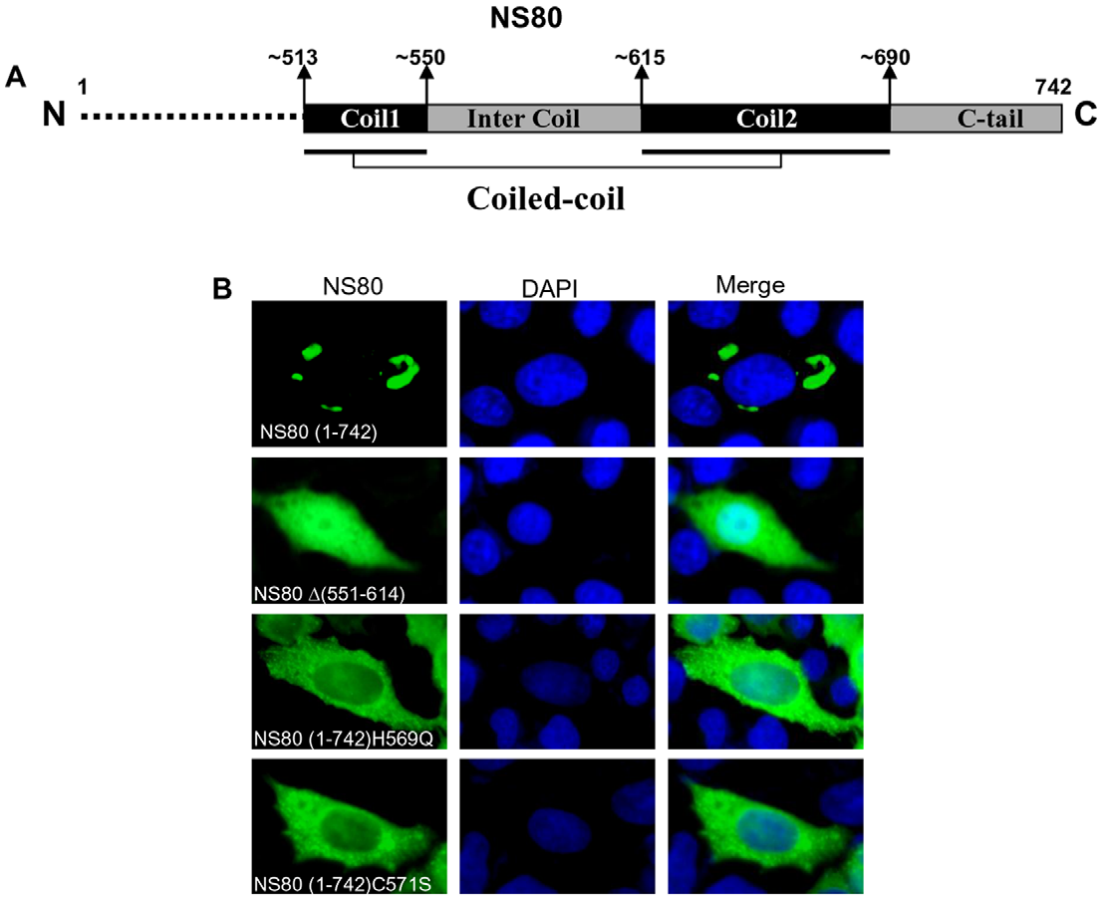
Mutation analyses of NS80 intercoil region. A . Schematic representation of aquareovirus NS80 C-terminal regions with four potential inclusion formation related domains. **B**. Immunofluorescence analysis of CIK cells transfected with plasmids expressing full-length NS80(1–742), deleted mutant NS80Δ(551–614) and point mutants NS80(1–742)H569Q, NS80(1–742)C571S. At 18 h p.t., cells were fixed and immunostained with corresponding antibodies. Nuclei were counterstained blue with DAPI.

Similar to MRV or ARV µNS, NS80 of aquareovirus also contains a conserved motif that may play a role in inclusion formation [12], [16], [34], which was predicted to be composed of two coils, the intercoil linker region and the C-tail (Fig. 10A). There are also two universally conserved residues His569 and Cys571 within the intercoil linker region (from residues 551 to 614). To determine whether the full length intercoil linker region or the residues His569 and Cys571 in NS80 is essential for inclusion formation, we constructed mutants with the whole linker region (551–614) deleted and generated single residue mutations by changing His569 to Gln or Cys571 to Ser. These mutants were designated as NS80Δ(551–614), NS80(1–742)H569Q and NS80(1–742)C571S. These mutants were expressed in Vero and CIK cells and analyzed using anti-NS80 antibodies and MAb FK2. IF observation indicated that the expressed mutants were uniformly distributed in the cytoplasm and the nucleus with dispersed pattern (Fig. 10B, image not shown for CIK cells), which was in completely contrary to the inclusion morphology formed by the wild type NS80 protein, demonstrating that the linker region 551–614 as well as His569 and Cys571 residues are all essential for forming inclusions.

### NS80 C-tail can be Replaced by Dimerized Protein GFP

**Figure 11:**
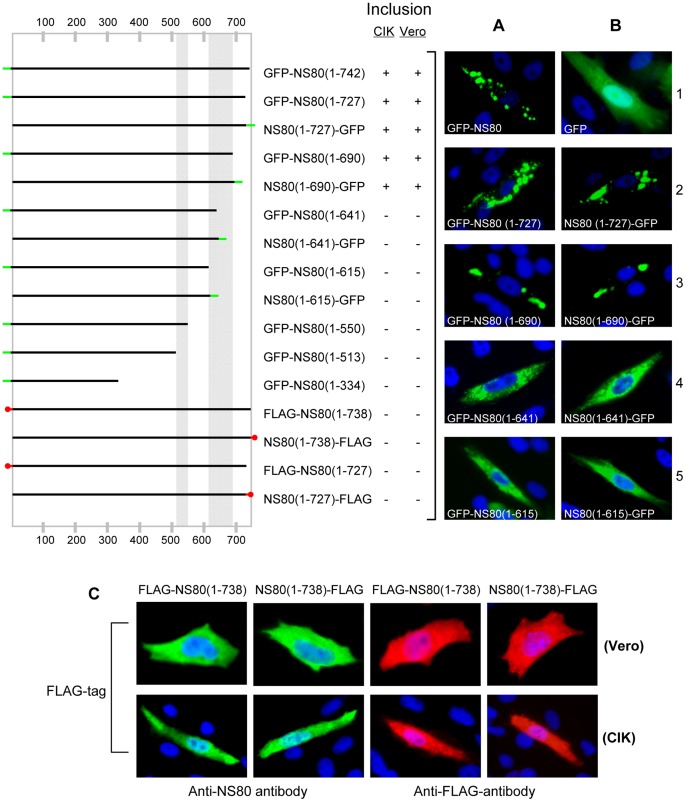
Intracellular distribution of GFP/FLAG-tagged NS80 C-terminal truncations. Left: Schematic representation of the capacity of GFP/FLAG tagged NS80 C-terminal truncations to form inclusions in transfected cells. Right: **A** and **B.** Fluorescence microscopy of GFP-tagged NS80 truncations. CIK cells were transfected with plasmids to express GFP-tagged NS80 C-terminal truncations either at the N- or C-terminus and then analyzed at 18 h p.t. by fluorescence microscopy. Bottom: **C.** IF microscopy of FLAG-tagged NS80 truncations. Vero or CIK cells were transfected with plasmids expressing FLAG-tag fused to the N- or C-terminus of NS80(1–738). At 18 h p.t., cells were fixed and immunostained with corresponding antibodies. Nuclei were stained blue with DAPI.

To better understand the role that the C-terminal region played in inclusion formation, we examined whether the C-tail of NS80 could be replaced with exogenous protein. As it has been known that GFP is a dimerized protein which can form coiled structures [24], [39], [40], to assess whether the C-terminus can be replaced by a dimeric protein, a panel of GFP tagged NS80 C-terminal truncations were generated and each of these plasmids was transfected into CIK and Vero cells for IB analysis with GFP-specific MAb (data not shown). Then, to determine the intracellular distribution of each protein, cells were detected with fluorescence microscopy. We observed that the GFP-tagged NS80 and GFP-tagged NS80 C-terminal tail truncations, such as GFP-NS80(1–727) and GFP-NS80(1–690), resulted in globular inclusions (Fig. 11A, row 1–3), similar to NS80(1–727)-GFP and NS80(1–690)-GFP (Fig. 11B, row 2–3). As a control, a non-fusion GFP showed diffusely distribution in the cytoplasm and the nucleus (Fig. 11B, row1), indicating that the C-tail could be replaced by GFP. However, with longer deletions of the coil2 region, a gradual phenotypic change from inclusion to diffused distribution occurred. Further complete deletion of the coil2 sequence from the C-terminus led to the complete loss of inclusion phenotype, whatever GFP tagged at N- or C-terminus, e.g. GFP-NS80(1–641), NS80(1–641)-GFP, GFP-NS80(1–615) and NS80(1–615)-GFP (Fig. 11A and B, row 4–5). These results indicate that the C-terminal tail can be substituted by dimeric GFP.

As a parallel comparison, we also performed examination using octapeptide FLAG as a non-dimeric tag [41] and constructed FLAG-tagged NS80 truncations. As it is shown in Fig. 11C that the FLAG-tagged NS80(1–738), either at N- or C-terminus (Image not shown for NS80(1–727)), showed no inclusion recovery in both transfected Vero and CIK cells, confirming that the C-tail of NS80 is involved in inclusion phenotype, and the epitope FLAG-tag can not functionally complement the structures. The results were summarized in Fig. 11 on the left panel.

## Discussion

Replication complexes of viruses in the family *Reorividae* contain dsRNA genomes, both viral structural and nonstructural proteins, complete or incomplete viral particles [6], [29], [42], [43]. The complex has been recognized to localize in specific structures in cytoplasm termed viral factory or viroplasm. Recent investigations on the nonstructural protein µNS in the genus *Orthoreovirus* and its analogue in other genus of the family *Reoviridea* indicated that this protein plays a central role in forming inclusion framework for stable viral life cycle [8], [15], [16], [28], [33], [44]. The results presented in this study suggested that aquareovirus NS80 protein is the crucial element for constructing aquareovirus inclusions and each of the C-terminal domains of NS80 is vital for establishing these structures.

In this study, we first demonstrated that the VF or inclusions structures can be detected by anti-NS80 antibody in both infected and transfected cells. Transfection and co-transfection experiments showed that single NS80 can induce cytoplasmic inclusions and recruit NS38 and GFP-tagged VP4 to these structures. Further co-IP experiments indicated that NS80 and NS38 could be immunoprecipitated mutually, demonstrating that NS80 and NS38 could interact with each other. It may need to note that a shorter polypeptide (about 70 kDa) of NS80 was also detected in infected cells in our current and previous investigation [35], suggesting that NS80 may be processed to a shorter form during viral infection. Indeed, the isoform of µNS was also detected in orthoreoviruses [10], [45], but the mechanisms that present µNS isoforms may be different in MRV and ARV [46]. Further work is needed to determine the interaction regions required for NS80 with other viral proteins, and define possible role of NS80 isoform in viral replication.

Next, we defined the regions essential for NS80 to form inclusions using deletion or truncation analysis. IF results indicated that the C-tail is indispensable for inclusion formation, since deletion of only 4 aa (SLLL, 739–742) from the C-terminal tail could result in the loss of inclusion phenotype. Besides, results of the truncations that lack N-terminal residues demonstrated that the C-terminal region ranging from residues 530 to 742 is likely the minimum sequences required for inclusion formation. This region contains 212 C-terminal amino acids, including the coiled-coil region and the C-tail. The length of this identified inclusion forming region is between identified µNS in MRV (471–721) [16] and ARV µNS (448–635) [15]. Further mutation analysis revealed that the complete linker region (551–614) as well as the conserved His and Cys residues within the intercoil motif are all required for inclusion formation.

To address whether the NS80 C-terminus can be replaced by exogenous proteins, the dimeric GFP was introduced to inclusion-deficient NS80 C-terminal truncations by tagging either at the N- or C-terminus. The results showed that the inclusion phenotype could be restored by tagging GFP with truncations lacking the C-tail sequences. However, this restoration can not be achieved with an epitope FLAG-tag. Together, these results strongly support the conclusion that NS80 can form dimer or basal small oligomer through self-interactions with the participation of its C-terminal region [17], [23].

The special finding in this study is that, with increasing deletion from the N-terminus, we observed a stepwise phenotype conversion from big condensed cytoplasmic to small nucleic inclusion, then to diffused intracellular distribution, whereas no similar transition pattern was reported in NS80 analogues in other reoviruses so far. Based on our observation of this phenomenon and bioinformatics analysis of no “NLS” being found within NS80, we suspected that the formation of these nucleic inclusions may have some relationship with the nucleus-associated factor β-catenin. Most notablely, nucleic inclusions were detected to colocalize with cellular protein β-catenin, but not ubiquitin, suggesting that the expression of some truncated NS80 lacking the N-terminal sequences may lead to the up regulation of expression of cellular protein β-catenin. Further studies are needed to address the molecular mechanism of this phenomenon and the relationships between viral inclusions and cellular proteins.

In addition, our experiments also showed that the size of viral factory or NS80-derived inclusions is not influenced by cytoskeletal protein tubulin because the treatment with microtubule-depolymerizing drug nocodazole did not prevent the formation of large inclusions. This result is supported by µNS of fusogenic orthoreovirus ARV and NS2 of Bluetongue Virus (BTV) because the globular inclusions or VIB-like structures formed by µNS in ARV and NS2 in BTV are not associated with microtubule network [15], [47]. Other homologous proteins such as MRV µNS need to interact with cytoskeletal proteins to form filamentous inclusions [10], [14]. These results may reflect different cellular mechanisms for inclusion formation related scaffolding protein in cytoplasmic trafficking from different viruses.

In summary, we found that single NS80 can form factory-like inclusions and can recruit NS38 or VP4 to its globular cytoplasmic inclusions, and the inclusion formation of aquareovirus did not rely on the microtubules network. In addition, we identified that NS80(530–742) is the minimal region required for aquareovirus NS80 inclusion formation, and two coils region, the linker region and the C-tail are all critical for these structure generation. Moreover, we found that the nuclear inclusion generated by the N-terminal deleted NS80 could colocalize with the cellular protein β-catenin. The results reported in this study not only revealed the function and functional regions of aquareovirus NS80 protein in inclusion formation, but also provided a new insight for further defining the relationships between viral inclusions and cellular proteins.

## Materials and Methods

### Cells and Viruses

Vero, Hela and L929 cells were obtained from the China Center for General Viruses Culture Collection (CCGVCC). The indicated three cell lines were maintained in monolayers in Dulbecco’s modified Eagle medium (DMEM, GIBCO) supplemented with 10% fetal bovine serum. CIK (*Ctenopharyngodon idellus kidney*) cells were grown at 28°C in Eagle’s minimum essential medium (MEM, Invitrogen) containing 2 mM L-glutamine, supplemented with 10% of fetal bovine serum [48]. GCRV-873 strain was isolated and stored in our laboratory and the propagation of the virus has been described previously [49].

### Antibodies

Rabbit or mouse polyclonal antibodies against GCRV-873 NS80 or NS38 protein were raised in our laboratory [35], [50]. All the prepared antibodies in our lab were titrated to optimize signal-to-noise ratios. The mouse monoclonal antibody (MAb) FK2, which recognize polyubiquitinylated and monoubiquitinylated proteins but not free ubiquitin, was purchased from Enzo Life Sciences, Inc. Mouse monoclonal anti-α-tubulin(clone B-5-1-2), anti-FLAG Tag antibody and alkaline-phosphatase-coupled goat anti-rabbit IgG or goat anti-mouse IgG were purchased from Sigma-Aldrich. Rabbit polyclonal antibody against β-catenin was the product of Abcam. FITC (fluorescein isothiocyanate)–conjugated goat anti-rabbit or goat anti-mouse immunoglobulin G (H+L) and Texas Red–conjugated goat anti-rabbit or goat anti-mouse immunoglobulin G (H+L) were purchased from Thermo Pierce.

### Plasmid Constructions

GCRV NS80 protein or its truncations as well as NS38 protein were expressed by cloning into mammalian expression vector pCI-neo (Promega, USA). The primers were designed based on GenBank sequences (AF403390, AF403395), and restriction enzyme digestion sites were introduced at 5′end of each primer pair. The construction of the recombinant plasmid expressing full-length NS80 protein (named as pCI-neo-NS80), was generated by using the prepared total cDNA of GCRV genome as template [35], [36]. To obtain plasmids expressing NS80 N- or C-terminal truncations, start or stop codons, and also restriction sites were introduced at different positions in S4 segment by PCR amplification. Each PCR product was digested with EcoRI and XbaI and then ligated to the pCI-neo vector which had been cut with the same enzymes. All the primers used for generating NS80 truncations are listed in the Table S1. To generate epitope FLAG-tagged fusions, the FLAG tag fusing to the N- or C-terminal of NS80 truncations were constructed by introducing the nucleotide sequence of the FLAG tag to the primers (Table S2). To express another nonstructural protein NS38, the recombinant pCI-neo-NS38 was generated by inserting PCR amplicon derived from the S9 segment into the EcoRI-XbaI site of pCI-neo vector. The sense primer was 5′-GAAGAATTCTGAGCTTACCGATTGACAA-3′ and the antisense primer was 5′-CATTCTAGAATAGCTCAGAGCGGCATG-3′. For each primer, the additional restriction enzyme site near the 5′ end was single underlined.

The pEGFP-C1 and pEGFP-N3 vectors (Clontech, USA) were used to generate constructs for the expression of *Aequorea victoria* enhanced green fluorescence protein (GFP) fusing to the N- or C-terminus of the desired NS80 regions. Fragments corresponding to NS80 truncations were obtained by PCR using specific primers with EcoRI and BamHI restriction sites incorporated at the desired position in the S4 gene. Each PCR-amplified fragment was cut with EcoRI and BamHI and ligated to pEGFP-C1 or pEGFP-N3. The recombinants pEGFP-C1-NS38 and pEGFP-C1-VP4 were generated by inserting PCR amplicon derived from the S9 or S5 segment into the EcoRI-BamHI or Hind III-BamHI (the isocaudamer of Bgl II) site of the pEGFP-C1 vector. All the primers used for generating GFP-fusions are listed in the Table S3. All restriction enzymes were obtained from Takara Bio Inc. The constructed plasmids were verified by sequencing (Invitrogen Biotechnology Inc, Shanghai, China).

The Site-directed Gene Mutagenesis Kit (Beyotime Company, China) was used according to the manufacturer’s protocol to generate the recombinant plasmids containing the point mutants including residues His569 changed to glutamine(Q) and Cys571 changed to serine(S), using pCI-neo-NS80 as template. The following mutagenic oligonucleotide primers were used: for the production of pCI-neo-NS80 H569Q, the forward primer was 5′-GTACCTCCACTCCCAAACGTGCGTCAATACC-3′ and the reverse primer was 5′-GGTATTGACGCACGTTTGGGAGTGGAGGTAC-3′; for the generation of pCI-neo-NS80 C571S, the forward primer was 5′-TCCACTCCCACACGTCCGTCAATACCCAGGAG-3′ and the reverse primer was 5′-CTCCTGGGTATTGACGGACGTGTGGGAGTGGA-3′. For each primer, the nucleotide change to give the desired amino acid change is double underlined. To construct the recombinant plasmid containing the deleted mutation with whole intercoil region (551 to 614), the pCI-neo-NS80 was used as a template for PCR amplification. The forward primer was 5′-CTCACCGCCCAGCTCTCCGACACCAT-3′ and the reverse primer was 5′-ATAGCTCTTGAGGTCCGAGATCGTTCGC-3′, both were 5′ phosphorylated. All constructs were confirmed by sequencing as indicated above.

### Infections and Nocodazole Treatment

Virus infection with CIK cells was performed as previously described [48]. Briefly, CIK cells were plated one or two days in advance and infected with 5 PFU/cell of GCRV-873 virus stock. After 45 min absorption, unabsorbed viruses were removed and cells were washed with PBS and further incubated with fresh MEM-2 (MEM with 2% FBS) medium. For nocodazole treatment, MEM-2 containing 10 µM nocodazole or without nocodazole were added, incubated at 28°C for 3 days, and the cultured viral lysates were titrated by using plaque assays [51]. In this study, the virus titers were obtained from two separated assays with three repeats.

### Transfections

Cells were seeded the day before transfection in 24-well plates. A total of 0.8 µg/well of each plasmid DNA was used for transfection according to the manufacturer’s instruction of Lipofectamine 2000 (Invitrogen Life Technologies). For co-transfection, the ratio of the plasmids used for transfection was with 1∶1 value unless otherwise stated. After 4–6 hours incubation, the complex was removed and fresh DMEM/MEM with 10% fetal bovine serum was added. Then cells were incubated at 37°C till visualizing using an Olympus-IX51 inverted microscope (for GFP fusion proteins) or processing for immunofluorescence microscopy (IF). The treatment of transfected cells with nocodazole was conducted as indicated above.

### IF Microscopy

Infected or transfected cells were fixed for 20 min at room temperature with 4% paraformaldehyde. Fixed cells were washed three times with phosphate-buffered saline (PBS) (137 mM NaCl, 3 mM KCl, 8 mM Na_2_HPO_4_, 1 mM KH_2_PO_4_ [pH 7.5]) and permeabilized for 10 min with 0.2% Triton X-100, followed by three times washing and blocking for 45 min at 37°C with 5% bovine serum albumin (BSA). Then, cells were incubated with primary antibodies at room temperature for 1 h. After that, three times washing were conducted and secondary antibodies were added. With incubation at room temperature for another 1 h, cells were washed three times with PBS and incubated with DAPI for 5 min. Finally, samples were examined with an Olympus-IX51 inverted fluorescence microscope and images were obtained and processed with Image-pro Express 6.0.

### Immunoblotting and Co-immunoprecipitation

For immunoblotting (IB) analysis, cell lysates were collected, pelleted and resuspended in PBS before resolved by SDS-PAGE. All samples in gels were transferred to polyvinylidene fluoride (PVDF) membranes by a semi-dry transfer cell (Bio-Rad, California, USA) for 45 min at 100 mA. Membranes were blocked by 3% BSA and then incubated with NS80 or NS38 specific antisera (1∶500) as primary antibodies. The alkaline-phosphatase-coupled goat anti-rabbit or goat anti-mouse IgG was used as the secondary antibodies (1∶2,000). Protein bands were detected by developing with NBT/BCIP AP substrate solution (Promega) according to the manufacturer’s instructions. For co-immunoprecipitation, infected or transfected cells were lysed by incubation for 5 to 10 min on ice in IP Lysis/Wash Buffer (0.025 M Tris, 0.15 M NaCl, 0.001 M EDTA, 1% NP-40, 5% glycerol; pH 7.4). The lysates were pre-cleared by incubation with 80 µl Control Agarose Resin slurry (40 µl of settled resin) at 4°C for 1 h and then incubated with the antibody-coupled resin by gently rocking at 4°C overnight. Immunoprecipitated proteins were washed for four times with 1×Modified Dulbecco’s PBS buffer, one time with 1×Conditioning Buffer and eluted by Elution Buffer. Then IB was conducted as described above to detect the desired protein band. All the reagents used in co-immunoprecipitation experiments were purchased from the Pierce (Thermo Scientific).

## Supporting Information

Table S1 **Construction of plasmids expressing NS80 or its truncations.**
(DOC)

Table S2 **Construction of plasmids expressing FLAG-tagged NS80 truncations.**
(DOC)

Table S3 **Construction of plasmids expressing GFP-tagged proteins.**
(DOC)
