# Supplementary material for: Aquareovirus NS80 Recruits Viral Proteins to Its Inclusions, and Its C-Terminal Domain Is the Primary Driving Force for Viral Inclusion Formation
Source: PLoS One. 2013 Feb 12;8(2):e55334. doi: 10.1371/journal.pone.0055334 (PMC3570539; doi:10.1371/journal.pone.0055334)
Supplement: Table S1 — Construction of plasmids expressing NS80 or its truncations. (DOC) [file pone.0055334.s001.doc]

**Table S1.Construction of plasmids expressing NS80 or its** truncations.

| **Construct**^a^ | **Primers(5’to 3’)^b^** | **Expressed protein^c^** | **Size(KDa)^d^** |
| --- | --- | --- | --- |
| pCI-neo-NS80(1-742) | F:GACGAATTCAAGAGCTTTGGAAGTAAC | NS80 | 79.6 |
|  | R:CATTCTAGAGACACAGAAACACAGAGC |  |  |
| pCI-neo-NS80(1-738) | F:GACGAATTCAAGAGCTTTGGAAGTAAC | NS80(1-738) | 79.2 |
|  | R:CATTCTAGATTAGGCAGGGTCGATGGC |  |  |
| pCI-neo-NS80(1-727) | F:GACGAATTCAAGAGCTTTGGAAGTAAC | NS80(1-727) | 78.1 |
|  | R:CATTCTAGATTAGGTGAGACCTGGCCCAAC |  |  |
| pCI-neo-NS80(1-700) | F:GACGAATTCAAGAGCTTTGGAAGTAAC | NS80(1-700) | 75.5 |
|  | R:CATTCTAGATTAAGCGGCGGCTTCGGCGGTAGC |  |  |
| pCI-neo-NS80(1-519) | F:GACGAATTCAAGAGCTTTGGAAGTAAC | NS80(1-519) | 55.7 |
|  | R:CAATCTAGATTAGAGTTCACGTAGGGTG |  |  |
| pCI-neo-NS80(1-129) | F:GACGAATTCAAGAGCTTTGGAAGTAAC | NS80(1-129) | 13.8 |
|  | R:CAATCTAGATTACAGAAGCACGAGTTCGC |  |  |
| pCI-neo-NS80(130-742) | F:AACGAATTCAACATGCGCTTTGCTGACCTG | NS80(130-742) | 65.8 |
|  | R:CATTCTAGAGACACAGAAACACAGAGC |  |  |
| pCI-neo-NS80(268-742) | F:TAAGAATTCAACATGCACATCGCTGATGACGAGAC | NS80(268-742) | 51.6 |
|  | R:CATTCTAGAGACACAGAAACACAGAGC |  |  |
| pCI-neo-NS80(335-742) | F:AATGAATTCAACATGTCCCTCCTTACCCTTCACAAC | NS80(335-742) | 44.1 |
|  | R:CATTCTAGAGACACAGAAACACAGAGC |  |  |
| pCI-neo-NS80(423-742) | F:AACGAATTCAACATGGTTATGATGAGACATGAT | NS80(423-742) | 34.5 |
|  | R:CATTCTAGAGACACAGAAACACAGAGC |  |  |
| pCI-neo-NS80(456-742) | F:GAAGAATTCAACATGTTAGCCCTCATGCAGATCG | NS80(456-742) | 30.7 |
|  | R:CATTCTAGAGACACAGAAACACAGAGC |  |  |
| pCI-neo-NS80(471-742) | F:TAAGAATTCAACATGGCCTGGCTTCGTGGCC | NS80(471-742) | 29.1 |
|  | R:CATTCTAGAGACACAGAAACACAGAGC |  |  |
| pCI-neo-NS80(485-742) | F:GAAGAATTCAACATGGCCACGACTGACTCCT | NS80(485-742) | 27.5 |
|  | R:CATTCTAGAGACACAGAAACACAGAGC |  |  |
| pCI-neo-NS80(496-742) | F:AATGAATTCAACATGGCCACCATCCTGGAAG | NS80(496-742) | 26.4 |
|  | R:CATTCTAGAGACACAGAAACACAGAGC |  |  |
| pCI-neo-NS80(502-742) | F:AATGAATTCAACATGGACAAGGGAATGCGCGT | NS80(502-742) | 25.8 |
|  | R:CATTCTAGAGACACAGAAACACAGAGC |  |  |
| pCI-neo-NS80(513-742) | F:GAAGAATTCAACATGTCCTCCACCCTACGTGA | NS80(513-742) | 24.7 |
|  | R:CATTCTAGAGACACAGAAACACAGAGC |  |  |
| pCI-neo-NS80(520-742) | F:GAAGAATTCAACATGGAGGCCTCCAACACCG | NS80(520-742) | 23.9 |
|  | R:CATTCTAGAGACACAGAAACACAGAGC |  |  |
| pCI-neo-NS80(525-742) | F:CAGGAATTCAACATGGCTCTTCAACGTCAGGTCAT | NS80(525-742) | 23.4 |
|  | R:CATTCTAGAGACACAGAAACACAGAGC |  |  |
| pCI-neo-NS80(530-742) | F:AAGGAATTCAACATGGTCATCGACATGGATGTGC | NS80(530-742) | 22.8 |
|  | R:CATTCTAGAGACACAGAAACACAGAGC |  |  |
| pCI-neo-NS80(534-742) | F:AATGAATTCAACATGGATGTGCAGATCAACGCG | NS80(534-742) | 22.4 |
|  | R:CATTCTAGAGACACAGAAACACAGAGC |  |  |
| pCI-neo-NS80(538-742) | F:AATGAATTCAACATGAACGCGCTGCTGCGAAC | NS80(538-742) | 21.9 |
|  | R:CATTCTAGAGACACAGAAACACAGAGC |  |  |
| pCI-neo-NS80(542-742) | F:AATGAATTCAACATGCGAACGATCTCGGACCT | NS80(542-742) | 21.5 |
|  | R:CATTCTAGAGACACAGAAACACAGAGC |  |  |
| pCI-neo-NS80(550-742) | F:CAGGAATTCAACATGTATACCAACCATCAGCAAG | NS80(550-742) | 20.6 |
|  | R:CATTCTAGAGACACAGAAACACAGAGC |  |  |
| pCI-neo-NS80(562-742) | F:CAGGAATTCAACATGATCCAACAGTACCTCCACT | NS80(562-742) | 19.2 |
|  | R:CATTCTAGAGACACAGAAACACAGAGC |  |  |
| pCI-neo-NS80(581-742) | F:GAAGAATTCAACATGCAAAGCGTAATGGGTGA | NS80(581-742) | 17.0 |
|  | R:CATTCTAGAGACACAGAAACACAGAGC |  |  |

**^a^** Each construct was designed to express the indicated amino acid residues of NS80.

**^b^** For each construct, forward primer (F) and reverse primer(R) are indicated. The added start codon was double underlined; and the added stop codon was underlined with a single wavy line; Restriction enzyme site EcoR I or Xba I added near the 5’ end of each primer was single underlined.

**^c^** Each construct was designed to express a truncated NS80 protein except the first one for full-length NS80.

**^d^** Predicted size of the expressed NS80 and its truncations.
