# Supplementary material for: Aquareovirus NS80 Recruits Viral Proteins to Its Inclusions, and Its C-Terminal Domain Is the Primary Driving Force for Viral Inclusion Formation
Source: PLoS One. 2013 Feb 12;8(2):e55334. doi: 10.1371/journal.pone.0055334 (PMC3570539; doi:10.1371/journal.pone.0055334)
Supplement: Table S2 — Construction of plasmids expressing FLAG-tagged NS80 truncations. (DOC) [file pone.0055334.s002.doc]

**Table S2. Construction of plasmids expressing FLAG-tagged NS80 truncation**s.

| **Construct**^a^ | **Primers(5’to 3’)^b^** |
| --- | --- |
| pCI-neo-FLAG-NS80(1-738) | F:GACGAATTCATGGACTACAAAGACGATGACGACAAGGCACGCCGCATTACTTT |
|  | R:CATTCTAGATTAGGCAGGGTCGATGGC |
| pCI-neo-NS80(1-738)-FLAG | F:GACGAATTCATGGCACGCCGCATTACTTTG |
|  | R:AATTCTAGATTACTTGTCGTCATCGTCTTTGTAGTCGGCAGGGTCGATGGCG |
| pCI-neo-FLAG-NS80(1-727) | F:GACGAATTCATGGACTACAAAGACGATGACGACAAGGCACGCCGCATTACTTT |
|  | R:GATTCTAGATTAGGTGAGACCTGGCCCAACGG |
| pCI-neo-NS80(1-727)-FLAG | F:GACGAATTCATGGCACGCCGCATTACTTTG |
|  | R:AATTCTAGATTACTTGTCGTCATCGTCTTTGTAGTCGGTGAGACCTGGCCCAA |

**^a^** Each construct was designed to express the indicated FLAG-tagged NS80 truncations.

**^b^** For each construct, forward primer (F) and reverse primer(R) were indicated. The added start codon was double underlined; and the added stop codon was underlined with a single wavy line; Restriction enzyme site EcoR I or Xba I added near the 5’ end of each primer was single underlined.
