# Supplementary material for: Aquareovirus NS80 Recruits Viral Proteins to Its Inclusions, and Its C-Terminal Domain Is the Primary Driving Force for Viral Inclusion Formation
Source: PLoS One. 2013 Feb 12;8(2):e55334. doi: 10.1371/journal.pone.0055334 (PMC3570539; doi:10.1371/journal.pone.0055334)
Supplement: Table S3 — Construction of plasmids expressing GFP-tagged proteins. (DOC) [file pone.0055334.s003.doc]

**Table S3. Construction of plasmids expressing GFP-tagged proteins**.

| **Construct^a^** | **Primers(5’to 3’)^b^** | **Expressed protein^c^** | **Size(KDa)^d^** |
| --- | --- | --- | --- |
| pEGFP-C1-NS80(1-742) | F:CATGAATTCTATGGCACGCCGCATTAC | GFP-NS80 | 108.3 |
|  | R:AATGGATCCCAGCAGCAGGGAGGCAG |  |  |
| pEGFP -C1-NS80(1-727) | F:CATGAATTCTATGGCACGCCGCATTAC | GFP-NS80(1-727) | 106.8 |
|  | R:AATGGATCCGGTGAGACCTGGCCCAA |  |  |
| pEGFP-C1-NS80(1-690) | F:CATGAATTCTATGGCACGCCGCATTAC | GFP-NS80(1-690) | 103.3 |
|  | R:TGCGGATCCCTGGAGTTGTTCATTGTCTT |  |  |
| pEGFP-C1-NS80(1-641) | F:CATGAATTCTATGGCACGCCGCATTAC | GFP-NS80(1-641) | 97.8 |
|  | R:AATGGATCCAGCGAGCTGAGCGGTCAGAT |  |  |
| pEGFP -C1-NS80(1-615) | F:CATGAATTCTATGGCACGCCGCATTAC | GFP-NS80(1-615) | 94.9 |
|  | R:AATGGATCCGAGAGGGGCAGTGACTTTAT |  |  |
| pEGFP -C1-NS80(1-550) | F:CATGAATTCTATGGCACGCCGCATTAC | GFP-NS80(1-550) | 87.8 |
|  | R:AATGGATCCATAGCTCTTGAGGTCCGAGAT |  |  |
| pEGFP-C1-NS80(1-513) | F:CATGAATTCTATGGCACGCCGCATTAC | GFP-NS80(1-513) | 83.6 |
|  | R:AATGGATCCGGAACCGGGAGTGTCAGCG |  |  |
| pEGFP-N3-NS80(1-727) | F:CATGAATTCTATGGCACGCCGCATTAC | NS80(1-727)-GFP | 105.5 |
|  | R:AATGGATCCGGTGAGACCTGGCCCAA |  |  |
| pEGFP-N3-NS80(1-690) | F:CATGAATTCTATGGCACGCCGCATTAC | NS80(1-690)-GFP | 102 |
|  | R:TGCGGATCCCTGGAGTTGTTCATTGTCTT |  |  |
| pEGFP-N3-NS80(1-641) | F:CATGAATTCTATGGCACGCCGCATTAC | NS80(1-641)-GFP | 96.5 |
|  | R:AATGGATCCAGCGAGCTGAGCGGTCAGAT |  |  |
| pEGFP-N3-NS80(1-615) | F:CATGAATTCTATGGCACGCCGCATTAC | NS80(1-615)-GFP | 93.6 |
|  | R:AATGGATCCGAGAGGGGCAGTGACTTTAT |  |  |
| pEGFP-C1-NS38 | F:AATGAATTCTATGGCACACACAGGCAC | GFP-NS38 | 66.4 |
|  | R:AATGGATCCCATACCCCCGATCGGCA |  |  |
| pEGFP-C1-VP4 | F:CGCAAGCTTACATGATCACCATTGTGGTT | GFP-VP4 | 108.9 |
|  | R: TAG AGATCT TCAAACCCCGGTCGAGGT |  |  |

**^a^** Each construct was designed to express a GFP-tagged fusions.

**^b^** The EcoR I or BamH I restriction enzyme site added near the 5’ end of each primer was single underlined. The added start codon of the forward primer was double underlined.

**^c^** In each of these protein, GFP was fused to the N terminus or C terminus of the indicated region.

**^d^** Predicted size of the expressed GFP fusion protein.
